# Magnetic resonance imaging in patients with cardiac implantable electronic devices

**DOI:** 10.1007/s12471-018-1192-3

**Published:** 2018-11-07

**Authors:** A. H. Maass, M. E. W. Hemels, C. P. Allaart

**Affiliations:** 1 Department of Cardiology, Thorax center, University of Groningen, University Medical Center Groningen, Groningen, The Netherlands; 2 Department of Cardiology, Rijnstate Hospital, Arnhem, The Netherlands; 3 Radboud University Hospital, University of Nijmegen, Nijmegen, The Netherlands; 4 Cardiology, Amsterdam Cardiovascular Sciences, Amsterdam UMC, Vrije Universiteit Amsterdam, Amsterdam, The Netherlands

**Keywords:** MRI, Pacemaker, ICD, Safety

## Abstract

In recent years the prevalence of implantation of a cardiac implantable electronic device (CIED) has increased due to expanding implantation indications and prolonged life expectancy. Diagnostic strategies increasingly employ magnetic resonance imaging (MRI) to aid therapeutic strategies. In earlier guidelines, MRI was contra-indicated in patients with CIEDs, mainly due to previous reports of severe complications. With the development of MRI-conditional CIEDs and recent evidence concerning non-MRI-conditional CIEDs, MRIs in CIED patients can be safely performed in many hospitals.

However, there are several questions that need to be addressed. Which patients can we scan? How can the scans be performed safely? And last but not least, can cardiac MRI provide diagnostic yield in patients with CIEDs?

Current European guidelines are rather outdated and vague about patient selection and practical issues. There are national guidelines on this topic but several issues need extra attention and those are addressed in this point of view. It is important to create an environment with proper patient selection without unnecessary MRI scans in CIED patients, but also without unnecessary fear of complications, preventing access to MRI in patients who can benefit from this powerful diagnostic tool.

## Introduction

In recent years the prevalence of implantation of a cardiac implantable electronic device (CIED) has increased [1] due to expanding implantation indications and prolonged life expectancy. This increase has been observed with both pacemakers and implantable cardioverter defibrillators (ICDs), as well as with implantable loop recorders. As pacemaker and ICD patients are prone to develop significant comorbidities, it is crucial that protocols for medical diagnostic and therapeutic procedures are established and followed to avoid temporary or permanent device malfunctioning (reviewed in [2]).

Diagnostic strategies increasingly employ magnetic resonance imaging (MRI). Over the last decades the number of MRI scans has doubled every 5 years [3]. It is estimated that 28% of CIED patients will have an indication for MRI scanning in a period of 4 years with one third of patients needing more than one scan [3]. In earlier guidelines, MRI was contra-indicated in patients with a CIED, mainly due to previous reports of severe complications. Major safety concerns were induction of arrhythmias, tip heating, mechanical concerns and electromagnetic effects[2].

With the development of MRI-conditional pacemakers in 2008, MRI-conditional ICDs in 2011, and recent evidence concerning non-MRI-conditional CIEDs, performing MRI scans in CIED patients has become part of daily routine in many hospitals. Whereas first-generation MRI-conditional devices had specially developed leads with a higher chance of peri-operative complications [4], presently most current leads and devices have an MRI-conditional label. In the latest European pacing guidelines, performing MRI in patients with MRI-conditional devices has received a Class IIa indication whereas non-MRI-conditional devices have received a Class IIb indication for MRI scanning [5]. Since the publication of these, rather outdated, guidelines several new publications increased the body of evidence concerning safety of MRI in patients with non-MRI-conditional devices. A fairly recent expert consensus statement of the Heart Rhythm Society provides extensive recommendations on several issues [6]. The Netherlands Society of Cardiology has also published practice guidelines surrounding MRI and CIEDs (https://www.nvvc.nl/media/richtlijn/237/Leidraad%20MRI.pdf).

A recent study confirms that the accumulating evidence of safe MRI in CIED patients has not yet translated into wide-spread access to MRI. Still, in patients with pacemakers, and even more so in patients with ICDs, MRI is denied by cardiologists or radiologists [7].

This point of view focuses on several practical issues: patient selection, considerations for programming and patient safety. Patients who might benefit from MRI should preferably not be denied access to this powerful diagnostic tool. We also discuss the possibility of performing cardiac MRI in patients with CIEDs as this is thought to be difficult, if not impossible, due to artefacts created by the metal-containing parts of the CIED system.

## Patient selection

Modern CIEDs from most manufacturers are labelled MRI-conditional. This concerns both pacemakers and ICDs, and includes modern techniques such as leadless pacemakers [8] and the subcutaneous ICD [9]. Several electrodes that have been on the market for quite some time also received the MRI-conditional label and when they are connected to a modern pacemaker or ICD from the same manufacturer, the whole system is MRI-conditional. Frequently, during device replacement or *de novo* implantations MRI-conditional components of several manufacturers are mixed and in that case the whole system cannot be labelled MRI-conditional.

MRI-conditional labelling is usually acquired by technical construction calculations and *in vitro* testing. Clinical patient data before achievement of the label were scarce but are accumulating after introduction into the market. For FDA licensing purposes, 2,629 CIED patients were prospectively followed and underwent a total of 872 MRI scans without any complications [3]. Clinical data are available in non-MRI-conditional systems as well. Currently, these data are robust with a very large prospective registry including 1,500 patients who underwent non-thoracic MRI in the MagnaSafe registry [10] and a recent meta-analysis which included 5,099 patients [11]. As yet, less data are available on the safety of MRI in patients with abandoned leads or epicardial leads. Even though several studies showed no serious adverse events in patients with abandoned leads [12, 13], sensitive markers of lead or tissue damage, such as changes in impedance or threshold, cannot be assessed. Other investigators found no problems with several issues posing a contraindication for MRI, such as batteries close to depletion and recall components [14]. Epicardial electrodes might pose a greater risk for use in the MRI environment since they have the disadvantage of not being cooled by blood flow. They were typically used in children and patients with congenital heart disease, but are nowadays increasingly used in patients who need cardiac resynchronisation therapy as well [15]. The lifetime chance of the need for MRI in young patients especially is very high and we urgently need data demonstrating the safety of MRI in patients with epicardial electrodes. The same holds true for patients with older, non-MRI-conditional coronary sinus electrodes.

## Practical issues around the MRI scan

**Fig. 1:**
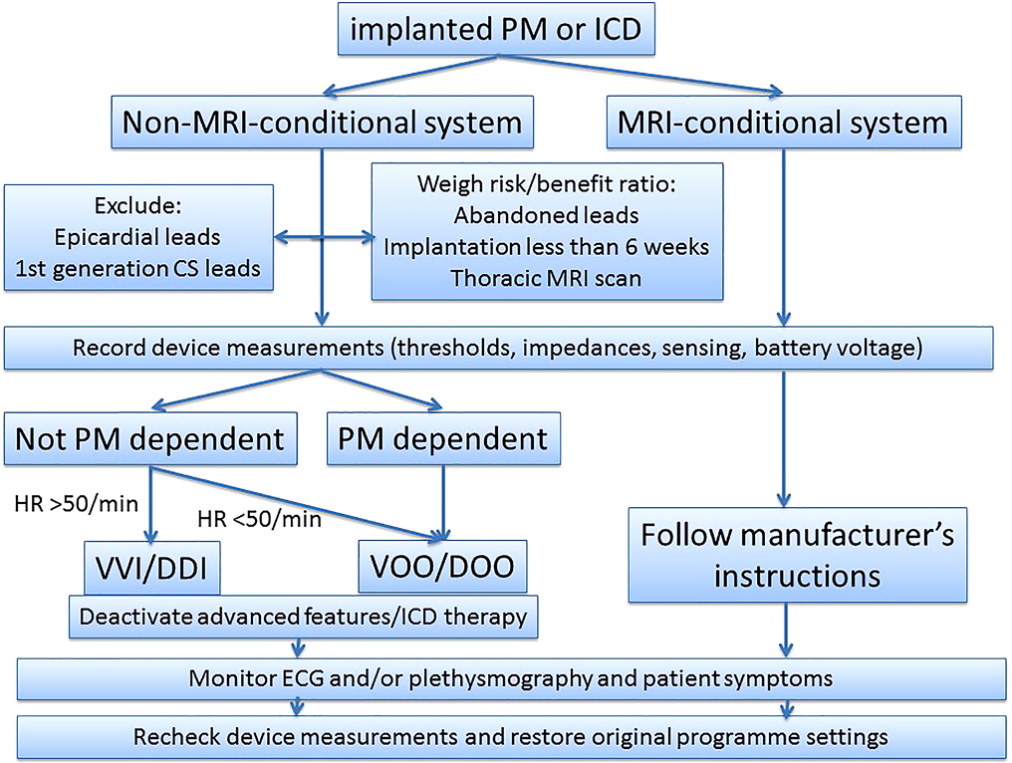
Flow diagram of patient selection and device programming for magnetic resonance imaging. *MRI* magnetic resonance imaging, *HR* heart rate, *PM* pacemaker, *ICD* implantable cardioverter defibrillator, *CS* coronary sinus, *ECG* electrocardiogram

To assure that a patient can be scanned safely, several steps have to be taken. These practical considerations evolve around the CIED system, but also involve MRI settings and patient monitoring during the scan (summarized in Fig. 1).

MRI scans in CIED patients require specific attention from the referring clinical department as well as the radiology and the cardiology departments. Therefore, as a first step, these labour-intensive scans should be reserved for patients that actually derive benefit, i. e. the scan should have clear therapeutic or prognostic implications that cannot be obtained by other diagnostic means. Before scheduling the patient this needs to be taken into account. Secondly, it needs to be determined whether the patient has an MRI-conditional or non-MIR-conditional system. According to manufacturer specifications of the MRI-conditional system, an MRI technical specialist should document the specific MRI conditions (e. g. Tesla, specific absorption rates, etcetera) (see Table 1). Thirdly, information has to be available about pacemaker dependency and recent technical measurements. Measurements should be within normal range and devices should not be near the end of their battery life given the chance of switching to a safe replacement mode during the scan.

**Table 1:** Technical considerations for MRI in patients with cardiac implantable electronic devices

Technical specification of magnetic resonance imaging
For MRI-conditional devices follow manufacturer’s instructions for scan
For non-MRI-conditional devices:
– 1.5 T MRI should be preferred
– Gradient slew rate should not exceed 200 T/m/s
– Dorsal patient position
– Imaging landmark near the device (thorax) should be avoided
– Local transmit coils should be avoided
– SAR and scan time should be limited
Emergency equipment/external defibrillator as well as a device programmer should be present during the MRI
Continuous patient monitoring (electrocardiography/pulse oximetry) during the MRI should be performed

*MRI* magnetic resonance imaging, *SAR* specific absorption rate

At the day of the scan, actual technical measurements should be performed and the device has to be reprogrammed according to manufacturer’s specifications in MRI-conditional devices. This specific programming is part of the conditions under which MRI is possible. In pacemaker-dependent patients an asynchronous mode should be programmed (VOO or DOO). In non-pacemaker-dependent patients a VVI of DDI mode should be programmed, however, in patients with high pacing percentages asynchronous pacing modes might be preferable. Additional advanced pacing features should be deactivated. In ICD patients all ICD therapies should be deactivated (see Table 2).

During the scan the patient should be monitored using electrocardiography (ECG) and/or plethysmography. The latter may be the preferable technique because ECG monitoring might be disturbed during the scan, whereas plethysmographic signals usually are not. However, plethysmography obviously remains sensitive to loss of contact if the patient is moving his fingers. Therefore, monitoring should be performed by a professional trained in haemodynamic monitoring who is also aware of the possible scenarios needing intervention (Table 3). ‘Power-on resets’ occur relatively frequently, especially in non-MRI-conditional devices leading to a change of pacing mode that is susceptible to oversensing [12]. An external defibrillator with external pacing capability should always be present in close proximity of the MRI scanner and the available personnel needs to be trained in its use. Currently, there is increasing evidence that magnet resonance scanning can also be performed without monitoring, especially in acute settings when monitoring is not readily available, due to the low rate of problems during the scan [16].

**Table 2:** Advice for programming devices in non-MRI-conditional patients undergoing magnetic resonance scanning

In pacemaker-dependent patients: asynchronous mode (VOO/DOO) with HF of 80–90/min with high output, RV-only pacing in biventricular devices
*In non-pacemaker-dependent patients:*
– Resting heart rate >50/min: VVI or DDI 30–40/min
– Resting heart rate <50/min: consider VOO/DOO 80–90/min with high output
– RV-only pacing in CRT patients
*Deactivate additional features:*
– Rate response mode
– Anti-tachycardia therapies (including anti-tachycardia pacing and shocks)—ICD only
– LV-triggered pacing (ventricular sense response)—biventricular devices only
– Anti-pacemaker-mediated tachycardia pacing (PMT algorithms)
– PVC-triggered pacing response
– PAC-triggered pacing response
– Atrial fibrillation therapies (rate smoothing, overdrive pacing, conducted atrial fibrillation response)
– Hysteresis pacing
– Magnet response (if the option exists)
– Noise response

*MRI* magnetic resonance imaging, *HF* heart frequency, *RV* right ventricular, *CRT* cardiac resynchronisation therapy, *ICD* implantable cardioverter defibrillator, *LV* left ventricular, *PMT* pacemaker-mediated tachycardia, *PVC* premature ventricular contraction, *PAC* premature atrial contraction

**Table 3:** Scenarios during magnetic resonance imaging requiring immediate action of the observing professional

Scenario	Possible reasons
Asystole	Device malfunction in asynchronous mode ‘Power-on reset’ with pacing inhibition by noise True bradycardia in non-pacemaker dependent patients in inhibitory mode
Ventricular fibrillation/tachycardia	R-on-T phenomenon in asynchronous pacing mode True arrhythmia
Sudden rise in heart rate	‘Power-on reset’ in inhibitory mode leading to pacing True endogenous tachycardia
Sudden drop in heart rate	‘Power-on reset’ in asynchronous mode True endogenous bradycardia in inhibitory mode

After the scan, the programme settings of the device should be restored to the pre-MRI settings and technical measurements should be performed. Special attention should be paid to changes in electrode impedances, sensing, pacing thresholds and battery measurements. In case of significant changes a prolonged period of observation should be considered.

## Cardiac MRI in patients with CIEDs

Non-invasive cardiac imaging is important for diagnostics [17] and planning of therapeutic interventions [18].

Previously, cardiac MRI was thought to be difficult, if not impossible, in patients with CIEDs due to artefacts created by the metal-containing parts of the CIED system.

Recent developments have shown that with specific MRI settings cardiac MRIs can be performed with reasonable quality. This is especially true in pacemaker implants in the right pectoral region and to a lesser degree in left-sided pacemakers and even less in ICDs due to the larger metal-containing battery. In non-MRI-conditional systems, thoracic MRIs have only partly been included in the large prospective registries but seem to be safe [19]. In this study, wideband pulse sequence for late gadolinium enhancement yields a high rate of studies unaffected by artefacts.

## Conclusions

MRI can be safely performed in both patients with MRI-conditional and non-MRI-conditional systems if specific programming is performed, technical limitations of magnetic resonance scanning are taken into consideration and patients are adequate monitored. Even cardiac MRI can be performed if the correct protocols are followed. Cardiologists and radiologists should be familiar with the protocols and not deny patients access to this powerful diagnostic tool.
